# Role of Nicotine Dependence in the Association between the *Dopamine Receptor* Gene *DRD3* and Major Depressive Disorder

**DOI:** 10.1371/journal.pone.0098199

**Published:** 2014-06-13

**Authors:** Tellervo Korhonen, Anu Loukola, Juho Wedenoja, Emma Nyman, Antti Latvala, Ulla Broms, Anja Häppölä, Tiina Paunio, Andrew J. Schrage, Jaqueline M. Vink, Hamdi Mbarek, Dorret I. Boomsma, Brenda W. J. H. Penninx, Michele L. Pergadia, Pamela A. F. Madden, Jaakko Kaprio

**Affiliations:** 1 Department of Public Health, Hjelt Institute, University of Helsinki, Helsinki, Finland; 2 Department of Mental Health and Substance Abuse Services, National Institute for Health and Welfare, Helsinki, Finland; 3 Institute of Public Health and Clinical Nutrition, University of Eastern Finland, Kuopio, Finland; 4 Institute for Molecular Medicine Finland FIMM, University of Helsinki, Helsinki, Finland; 5 Department of Psychiatry, Helsinki University Central Hospital, Helsinki, Finland; 6 Washington University School of Medicine, Department of Psychiatry, Saint Louis, Michigan, United States of America; 7 Department of Biological Psychology/Netherlands Twin Register, VU University, Amsterdam, The Netherlands; 8 Department of Psychiatry, VU University Medical Center/GGZ InGeest, Amsterdam, The Netherlands; Yale University, United States of America

## Abstract

**Background:**

The aims of this study were to analyze associations of *dopamine receptor* genes (*DRD1-5*) with Major Depressive Disorder (MDD) and nicotine dependence (ND), and to investigate whether ND moderates genetic influences on MDD.

**Methods:**

The sample was ascertained from the Finnish Twin Cohort. Twin pairs concordant for smoking history were recruited along with their family members, as part of the multisite Nicotine Addiction Genetics consortium. Genetic association analyses were based on 1428 adults. Total of 70 tagging single nucleotide polymorphisms within the *dopamine receptor* genes were genotyped and analyzed for association with MDD, ND, and MD-ND co-morbidity. Individual level logistic regression analyses were based on 1296 adults with data on ND and MDD diagnoses, as well as on *dopamine receptor* genotypes adjusted for sex, age, and alcohol use. Four independent samples, such as population-based and case-control samples, were used for replication.

**Results:**

Rs2399496, located 1.5 kb downstream of *DRD3*, showed suggestive association for MDD (p = 0.00076) and significant association for MDD-ND co-morbidity (p = 0.000079). Suggestive gene-(rs2399496) by-ND-interaction justified analyses by genetic risk variant and ND status. Individuals with ND and two minor alleles (AA) of rs2399496 had almost six-fold risk for MDD (OR 5.74, 95%CI 3.12–10.5, p = 9.010e-09) compared to individuals without ND and with two major alleles (TT).

**Conclusions:**

Significant association between a variant downstream of *DRD3* and a co-morbid MDD-ND phenotype was detected. Our results further suggest that nicotine dependence may potentiate the influence of the *DRD3* genetic variant on MDD.

## Introduction

Depression, ranging from mild depressed mood to major depressive disorder (MDD) [1] is estimated to be the second leading cause of disability worldwide by 2020 [2]. Approximately 8–13% of the general population experience clinical depression during their lifetime [3]. Persistent smoking, being primarily sustained by nicotine dependence (ND), represents one of the most preventable causes of morbidity and mortality. Estimated prevalence of ND among Finnish ever smokers is 48–52% [4]. Depression is known to co-occur with smoking and ND [5]. While this association is well established, causal influences may be posited under several hypotheses. Twin and family studies show significant genetic correlations suggesting that shared genetic predisposition underlies this co-occurrence [6]. Genes are estimated to explain about 40% of variability in risk of developing MDD [7], and 40–75% in etiology of ND [6]. Genome-wide association (GWA) studies and meta-analyses show robust association between the *CHRNA5-CHRNA3-CHRNB4* nicotinic acetylcholine receptor gene cluster on chromosome 15q25 and smoking phenotypes including ND [8]. However, the identified variants do not explain the extent of familial variation for ND. Furthermore, although the *CHRNA5-CHRNA3-CHRNB4* cluster has been associated with many ND phenotypes, to our knowledge, it has not been directly associated with MDD, although the rs11636753 in *CHRNB4* showed suggestive association with the co-morbidity of MDD and ND [4].

Dopamine receptor genes may also be of interest to explain the association between MDD and ND. Deficiency in dopaminergic neurotransmission may underlie MDD symptomatology [2] and dysfunctional mesolimbic dopamine system plausibly underlies substance dependence [3]. Nicotinic acetylcholine receptors are widely distributed in mesolimbic reward pathways. Thus, nicotine can increase extracellular dopamine levels in these reward pathways [2]. Candidate gene studies have implicated dopaminergic pathway genes in depression and ND. The role of *DRD2* and *ANKK1* variants in smoking and ND has been suggested in various populations [9]–[13]. Variation in *DRD2* has been associated with depressiveness [14]. Evidence exists also for involvement of *DRD4* in mood disorders [15] and ND [16].

Genotype-by-phenotype-interactions concerning smoking and depression have been sparsely investigated, with a few small-scale studies focusing on *dopamine receptor* genes. A significant *DRD4* genotype-by-depression-interaction was found for stimulation- and negative-affect-reduction-smoking [17]. Likelihood of increased smoking level rose 2-fold with each additional *DRD2/ANKK1* rs1800497 minor allele, with a pronounced association among adolescents with depression symptoms [18]. Further, the association between smoking cessation and lifetime depression was significantly modified by *DRD2/ANKK1* rs1800497 genotype [19]. Given the co-occurrence and plausible shared etiology of ND and depression, it is pertinent to investigate whether *dopamine receptor* gene variants have pleiotropic associations on ND and MDD and whether the association between *dopamine receptor* genes and ND is modified by MDD - or *vice versa*.

We aimed to investigate: 1) magnitude of shared genetic factors underlying the association between lifetime DSM-IV diagnoses of MDD and ND; 2) whether ND moderates genetic influences on MDD or *vice versa*; 3) dopamine receptor genes’ single nucleotide polymorphisms (SNPs) for associations with MDD and ND. Finally, we tested two alternative hypotheses: A) MDD modifies the association between dopamine receptor genotype and ND; B) ND modifies the association between dopamine receptor genotype and MDD.

## Methods

### Ethics Statement

The authors assert that all procedures contributing to this work comply with the ethical standards of the relevant national and institutional committees on human experimentation and with the Helsinki Declaration of 1975, as revised in 2008.

### Sample

Sample collection has been previously described in detail [20], [21]. It was ascertained from the Finnish Twin Cohort of adult twins born in 1938–1957. Based on earlier questionnaires, ever-smoking concordant twin pairs and their family members were recruited in 2001–2005 for the Nicotine Addiction Genetics (NAG) Finland study, as part of the consortium including Finland, Australia, and USA. Data from diagnostic interview, blood samples, and informed consent were available on 2188 individuals. The study was approved by the Ethics committee of the Hospital District of Helsinki and Uusimaa, Finland and by the IRB of Washington University, St. Louis, Missouri, USA.

The quantitative genetic models included 115 MZ and 415 DZ pairs. The genetic association analyses included 1428 individuals (1128 twins, 271 siblings, 29 other family members; mean age 55.6 years, 59% males) who smoked on average 19.7 (SD 9.9) cigarettes per day (CPD). Ninety four percent had smoked ≥100 cigarettes over lifetime, 51.5% fulfilling the ND DSM-IV criteria. Prevalence of DSM-IV MDD was 17.2% (Table 1A). Multiple logistic regression analyses included 1296 individuals (mean age 55.2 years, 60.6% males). All had smoked ≥100 cigarettes over lifetime the average CPD being 18.9 (SD 10.4). ND and MDD prevalence was 54.5% and 18.4%, respectively (Table 1B).

**Table 1:** Basic characteristics of the study samples used in (A) genetic association analyses (N = 1428) and (B) logistic regressions (N = 1296).

A	Number of families	Number of individuals	MZ twins	DZ twins	Regular smoker	CPD ≥20	DSM-IV ND *	DSM-IV MDD	Number of DSM-IV MDD symptoms: min-max (mean)	Co-morbidity of MDD and ND	Mean age (years)
Total	735	1428	140	970	1346	649	735	246	0–9 (1.6)	173	55.6
					(94.3%)	(45.4%)	(51.5%)	(17.2%)	(SD 2.6)	(12.1%)	
Males a		845	98	589	817	456	440	104	0–9 (1.2)	73	55.2
		(59%)			(96.7%)	(54.0%)	(52.1%)	(12.3%)	(SD 2.5)	(8.6%)	
Females ^b^		583	42	381	529	193	295	142	0–9 (2.3)	100	56.1
		(41%)			(90.7%)	(33.1%)	(50.6%)	(24.4%)	(SD 3.1)	(17.2%)	

B	Number of families	Number of individuals	MZ twins	DZ twins	Regular smoker	CPD ≥20	DSM-IV ND *	DSM-IV MDD	Number of DSM-IV MDD symptoms: min-max (mean)	Co-morbidity of MDD and ND	Mean age (years)
Total	709	1296	129	888	1243	598	678	238	0–9 (1.6)	166	55.2
					(95.9%)	(48.1%)	(54.5%)	(18.4%)	(SD 2.8)	(12.8%)	
Males^ c^		785	90	542	765	426	413	102	0–9 (1.2)	72	55.0
		(61%)			(97.5%)	(55.8%)	(54.0%)	(13.0%)	(SD 2.5)	(9.2%)	
Females^ d^		511	39	346	478	172	265	136	0–9 (2.3)	94	55.4
		(39%)			(93.4%)	(36.0%)	(55.4%)	(26.6%)	(SD 3.1)	(18.4%)	

a , 74 with unconfirmed zygosity; ^b^, 34 with unconfirmed zygosity; ^c^ 44 with unconfirmed zygosity; ^d^ 23 with unconfirmed zygosity: *, Conditioned for regular smoking.

MZ, monozygotic; DZ, dizygotic; CPD, cigarettes per day; DSM-IV, Diagnostic and Statistical Manual of Mental Disorders, 4^th^ edition (APA 1994); MDD, major depressive disorder; ND, nicotine dependence.

### Phenotypes

Participants were interviewed using the diagnostic Semi-Structured Assessment for the Genetics of Alcoholism (SSAGA) [22] protocol including an additional section on smoking behavior and ND adapted from the Composite International Diagnostic Interview (CIDI) [23]. The following phenotypes were used: DSM-IV diagnosis of lifetime MDD (presence of depressed mood, irritable mood when age <18 years or diminished interest or pleasure in activities; altogether ≥5 symptoms out of 9 symptoms of depression clustering within 2 weeks leading to impairment in social, occupational or other important functioning), number of DSM-IV MDD symptoms, DSM-IV diagnosis of ND (≥3 symptoms out of 7, occurring within a year), number of DSM-IV ND symptoms, and the binary phenotype of co-morbidity of MDD and ND. (Phenotype correlations are presented in Table S1 in File S1). In hypothesis testing sex, age and alcohol use (defined as number of binge drinking days per year, binge drinking meaning ≥5 drinks at one occasion) were considered as potential confounders. In post-hoc analyses heavy smoking (≥20 CPD during heaviest smoking period or ≥40 cigarettes in a single day) was used.

### Genotyping

DNA was extracted from blood samples by standard methods. Altogether 303 individuals were genotyped for 76 SNPs in all known dopamine receptor genes (*DRD1-5*) using Sequenom’s homogeneous hME and iPLEX Gold technology (Sequenom, San Diego, CA, USA), as previously described [4]. For 1125 individuals, genotypes were derived from GWA data. Of the 76 SNPs genotyped with Sequenom, 70 were available in the GWA data (21 *DRD1* SNPs, 30 *DRD2*/*ANKK1* SNPs, 15 *DRD3* SNPs, two *DRD4* SNPs, and two *DRD5* SNPs). All analyses in this paper are based on these 70 SNPs. Genotyping was performed at the Welcome Trust Sanger Institute (Hinxton, UK) on the Human670-QuadCustom Illumina BeadChip (Illumina, Inc., San Diego, CA, USA), as previously described [4]. Altogether 29 markers were genotyped, 41 being imputed using IMPUTE v2.1.0 [24] using HapMap rel#24 CEU - NCBI Build 36 (dbSNP b126) as reference panel. The reference panel used in the imputation was HapMap rel#24 CEU - NCBI Build 36 (dbSNP b126). The posterior probability threshold for "best-guess" imputed genotype was 0.9: genotypes below the threshold were set to missing. Marker quality controls are presented in Table S2 in File S1.

### Statistical Analyses

#### Logistic Regressions

To verify the expected association between lifetime MDD and ND, individual level logistic regressions were applied for the affected/non-affected phenotypes adjusted for sex, age, and alcohol use, using the Stata 11.1 statistical software 1 [25]. Since observations on members within family may be correlated, this dependence (i.e. lack of statistical independence of individual observations due to genetic and familial factors) was statistically accounted for by using robust estimators of variance and the cluster option when estimating standard errors [26].

#### Quantitative Genetic Modeling

The quantitative genetic models included 115 MZ and 415 DZ twin pairs. A bivariate Cholesky decomposition for number of MDD and ND symptoms was conducted to estimate the genetic and environmental correlations underlying the phenotypic association. Univariate moderation models were conducted to examine whether the number of ND symptoms moderates the magnitude of genetic or environmental variance of MDD symptoms, and *vice versa*. This model extends the standard univariate twin model by adding a moderator effect, *β*, on the estimated additive genetic, common environmental and unique environmental paths of the model. A *β* coefficient that differs significantly from zero is regarded as evidence for a moderating effect on the genetic or environmental path in question. The model takes into account the phenotypic association between the two traits [27]. The modeling was conducted with the statistical package Mx, using standard Mx scripts (http://www.psy.vu.nl/mxbib/).

#### Linkage Disequilibrium Analyses

The linkage disequilibrium (LD) between SNPs was estimated among non-related individuals (one per family) by using Haploview 4.2 [28]. Haplotype blocks were defined according to the 'solid spine of LD' algorithm by using the default threshold values for block estimation.

#### Genetic Association Analyses

Qualitative association analyses were performed with Pseudomarker [29], which performs separate and joint linkage and LD analyses, testing each marker locus against a phenotype-based 'pseudomarker' locus. This likelihood-based estimation method is numerically equivalent to model-free analysis, and efficiently uses data on all family types. Both recessive and dominant models (default parameters) were fitted. Additive model could not be tested as it is not implemented in Pseudomarker. P-values were minimized over 'LD given linkage', 'LD given no linkage', and 'LD and linkage' (joint test), as well as dominant and recessive models. Quantitative association analysis was performed with QTDT [30] with sex and age at recruitment as covariates. In the analysis the proportion of alleles shared identically by descent (IBD) were estimated by multipoint computation of MERLIN [31] to extract maximal inheritance information from the pedigrees. The total association model was used, allowing powerful analysis of the sample including incomplete families. In the analysis, the variance components 'polygenic', 'non-shared environment' (environmental effects unique to each family member), 'common environment' (environmental effects shared by all related individuals), 'nuclear family environment' (environmental effects shared by all members of a nuclear family), and 'twin environment' (environmental effects shared only by twins) were used to model the phenotypic similarities between related individuals.

#### Hypothesis Testing

For testing the study hypotheses, we conducted logistic regressions to analyze the effect size and significance of rs2399496 coded 0 (TT = 0 minor alleles), 1 (TA = one minor allele), and 2 (AA = two minor alleles). We used the recessive model as the previous genetic association analyses produced the best results on this gene when using a recessive model. When testing the hypothesis (A) ‘Genetic vulnerability potentiated by self-medication’ the outcome was binary ND, while the assumed modifying variable was MDD. Gene-by-MDD- interaction was tested using the Nested Likelihood-ratio approach. When testing the hypothesis (B) ‘Genetic vulnerability potentiated by chronic exposure to risk factor’ the outcome was binary MDD, while the assumed modifying variable was ND. Similarly, gene-by-ND-interaction was tested using the Nested Likelihood-ratio approach. Logistic regression analyses were adjusted for sex, age, and alcohol use and clustering by family number option was applied [26].

#### Accounting for Multiple Testing

To account for multiple testing we used a modified Bonferroni correction to set p-value thresholds for significant and suggestive association. As the analyzed markers and traits are correlated, the number of independent markers and traits was estimated with SNPSpD and matSpD [32], respectively, and their MeffLi and VeffLi estimates [33] were used as they were smaller than Meff and Veff, respectively, as recommended by the author (http://gump.qimr.edu.au/general/daleN/SNPSpD/). In our data set, the number of independent markers was 36.9, and the number of independent traits was 3.20. A p-value threshold of 0.00042 for significant association was achieved by dividing p = 0.05 by the product of the number of independent markers and the number of independent traits. A p-value threshold of 0.0014 for suggestive association was achieved by dividing p = 0.05 by the number of independent markers.

### Replication

In an attempt to replicate the detected association, we analyzed the top-three SNPs (rs2399496, rs3732790, and rs2134655) in four independent data sets as follows: a Finnish adolescent twin sample [FT12: N = 967, DSM-IV MDD and Fagerström Nicotine Dependence Test (FTND) available], a Finnish adult population sample of unrelated individuals [Health2000: N = 2123, CIDI for major depressive episode, Beck Depression Inventory (BDI), smoking status, and CPD available], an Australian twin family sample (NAG-OZALC: N = 4425, DSM-IV MDD and DSM-IV ND available], and a Dutch sample combining data of the Netherlands Twin Register (NTR) and Study of Depression and Anxiety (NESDA) studies (NTR-NESDA: N = 1613 MDD cases, N = 1661 controls, DSM-IV MDD, FTND, and ever smoking available). These replication datasets are described in detail earlier [34]–[39].

In two of the replication samples, FT12 and the Health2000, the analyses were performed respectively with the study sample. In the NAG-AUS replication sample analyses were performed using MQLS (http://www.sph.umich.edu/csg/liang/MQLS/) [40] for the binary traits and MERLIN (http://www.sph.umich.edu/csg/abecasis/Merlin/index.html) [31] or MERLIN Offline for the continuous variables. Finally, in the Dutch NTR-NESDA replication sample analyses were performed using PLINK (http://pngu.mgh.harvard.edu/purcell/plink/) [41] logistic regression adjusting for age, sex, and principal components to correct for population stratification. The SNP associations with MDD were first tested in the whole sample, and then restricted to ever smokers. Next, SNP associations with ND were tested among smokers with data on FTND (cases defined as FTND≥4, controls defined as FTND 0–3). Finally, SNP associations with MDD-ND co-morbidity were tested among smokers with data on FTND. Genotype data for three SNPs in *DRD3* (rs2399496, rs3732790, and rs2134655, or correlates of those) were derived from GWA data. We used the recessive model in this replication analysis.

## Results

As expected, ND and MDD were significantly associated: individuals with MDD diagnosis having higher likelihood for lifetime ND (adjusted OR = 2.63, 95%CI 1.94–3.58, p = 3.0e-10) and individuals with ND diagnosis having higher likelihood for lifetime MDD (adjusted OR = 2.51, 95%CI 1.83–3.46, p = 7.6e-09).

The bivariate quantitative twin model on MDD and ND symptoms indicated substantial, although non-significant, correlation between genetic components (r_A_ = 0.51, 95% CI −0.11, +1.00), whereas the correlation between environmental components was moderate (r_E_ = 0.21, 95% CI 0.09, +0.32). Albeit wide confidence intervals, these results suggested that a substantial proportion of the correlation between ND and MDD may derive from shared genetic factors, justifying further analyses of specific genes. The univariate moderation models indicated that ND symptoms did not significantly moderate additive genetic (χ^2^(1) = 2.05, p = 0.15) or common environmental (χ^2^(1)<0.01, p>0.99) variance of MDD. Instead, moderating on unique environmental variance was detected (χ^2^(1) = 14.0, p<0.001), such that this variance (including error variance) of MDD symptoms increased 5.3-fold in the absence of ND. No significant moderation by MDD symptoms for genetic or environmental variance of ND appeared (χ^2^(3) = 0.36, p = 0.95).

The LD blocks for the SNPs were similar to those in the HapMap CEPH data (Figure S1 in File S1) and the somewhat stronger intermarker LD is in agreement with previous findings from the Finnish population [42]. We detected a significant association between *DRD3* rs2399496 and the co-morbid phenotype of MDD and ND (p = 0.000079). Rs2399496 also showed suggestive association with MDD (p = 0.00076) and a similar trend with MDD symptoms (p = 0.0017). No significant or suggestive association for ND diagnosis or symptoms appeared. We detected no significant or suggestive association with SNPs in the other genes (Table S3 in File S1). Association results for *DRD3* SNPs are presented in Table 2.

**Table 2:** Association analyses results (p-values) for *DRD3* SNPs.

Marker	Gene	DSM-IV MDD	DSM-IV MDD symptoms	DSM-IV ND	DSM-IV ND symptoms	Co-morbidity of DSM-IV MDD and ND
rs2399496	*DRD3*	**0.00076** b	0.0017	0.058	1.000	**0.000079** a
rs3732790	*DRD3*	0.0087	0.0072	0.482	0.286	0.011
rs2134655	*DRD3*	0.125	0.037	0.696	0.483	0.047
rs9880168	*DRD3*	0.072	0.337	0.996	0.584	0.194
rs324036	*DRD3*	0.098	0.337	1.000	1.000	0.089
rs324035	*DRD3*	0.759	0.732	0.533	0.695	0.910
rs167771	*DRD3*	0.998	0.609	0.985	0.901	0.648
rs11721264	*DRD3*	0.869	0.529	0.330	0.464	0.828
rs226082	*DRD3*	0.674	0.465	0.445	0.460	0.950
rs324029	*DRD3*	0.720	0.381	0.383	0.437	0.874
rs16822416	*DRD3*	0.304	0.876	0.740	0.674	0.115
rs9825563	*DRD3*	0.128	0.311	0.998	0.629	0.209
rs7629232	*DRD3*	0.207	0.289	0.423	0.915	0.333
rs6787134	*DRD3*	0.947	0.938	0.944	0.678	0.952
rs1354348	*DRD3*	0.292	0.678	0.592	0.035	0.556

DSM-IV, Diagnostic and Statistical Manual of Mental Disorders, 4^th^ edition (APA 1994); MDD, major depressive disorder; ND, nicotine dependence.

a , significant p-value (study-specific threshold p<0.00042).

b , suggestive p-value (study-specific threshold 0.00042<p<0.0014).

To follow up the marker exhibiting significant association for MDD-ND co-morbidity (rs2399496 in *DRD3*), we divided individuals with rs2399496 genotype available (N = 1353) into those fulfilling (N = 692) and not fulfilling (N = 661) the DSM-IV ND criteria. In separate association analyses for MDD, the association signal emerged solely from nicotine dependent subjects (data not shown). Similarly, we divided individuals into those fulfilling (N = 239) and not fulfilling (N = 1114) the MDD criteria and separately performed association analyses for ND, both subgroups giving negative results (data not shown).

We could not replicate the association between rs2399496 and depression or the co-morbid phenotype in the adolescent sample (FT12), in the Finnish population sample (Health2000) or in the Australian twin family sample (NAG-OZALC; rs9817063 used as proxy for rs2399496, intermarker r^2^ =  0.8–0.9, depending on the reference sample). Best evidence for replication was seen in the NTR-NESDA sample for rs3732790 which is in high LD with rs2399496 (r^2^ = 0.67, D’ = 0.997). No significant association with MDD was seen in the whole NTR-NESDA sample (OR = 1.13, 95%CI 0.93, 1.36, p = 0.21) or among ever smokers (OR = 1.19, 95%CI 0.94, 1.52, p = 0.15). However, when analyzing the co-morbid phenotype of MDD and ND the association became stronger and significant (OR = 1.56, 95% CI 1.05, 2.33, p = 0.03). Consistently with the study sample, no statistically significant association was seen between rs3732790 and ND (OR = 1.17, 95%CI 0.88, 1.55, p = 0.28). Association results for all replication samples are presented in detail in Tables S4a, S4b, S4c, and S4d in File S1.

Finally, two hypotheses were tested in the study sample, *i.e.* whether MDD potentiates the association of the SNP (rs2399496) with ND or *vice versa*. When the SNP’s association with ND was adjusted for MDD, sex, age, and alcohol use individuals carrying one (TA) or two (AA) minor alleles did not have significantly elevated risk for lifetime ND when compared to individuals homozygous for the major allele (TT). Thus, the first hypothesis was rejected. When the SNP’s association with MDD was adjusted for ND, sex, age, and alcohol use, individuals carrying two minor alleles (AA) had a nearly 2-fold risk for lifetime MDD (OR 1.89, 95% CI 1.26–2.84, p = 0.002) compared to individuals homozygous for the major allele (TT) (Table 3). Although the interaction test using the Nested Likelihood-ratio approach showed only a trend towards SNP (rs2399496)-by-ND-interaction (LR χ^2^(2) = 5.13, p = 0.08) the p-value was <0.10 – a cut point often used to perform additional analyses. Thus, the analyses separately by ND status were justified. Among nicotine dependent subjects (N = 678), the corresponding genetic risk for MDD was 2.29-fold (95% CI 1.37–3.83, p = 0.002), while no association was seen among non-dependent subjects (N = 618) (Table 4).

**Table 3:** Logistic regressions on the associations of lifetime DSM-IV nicotine dependence (ND)^a^ and the *DRD3* variant rs2399496 with lifetime DSM-IV major depressive disorder (MDD) b (N = 1296).

Outcome	Model 1^c^			Model 2		
MDD	Independent association of ND and gene			Associations of ND and gene adjusted for each other^c^		
Risk Factor	OR	95% CI	p-value	OR	95% CI	p-value
ND						
No	1.00			1.00		
Yes	2.51	1.83–3.46	7.569e–09	2.48	1.80–3.41	1.512e–08
rs2399496						
TT	1.00			1.00		
TA	1.32	0.92–1.88	0.130	1.28	0.89–1.83	0.185
AA	1.97	1.30–2.96	0.001	1.89	1.26–2.84	0.002

a DSM-IV nicotine dependence diagnosis.

b DSM-IV major depression disorder diagnosis.

c Adjusted for sex, age, and alcohol abuse.

**Table 4:** Logistic regressions on the associations of the *DRD3* variant rs2399496 with DSM-IV major depressive disorder (MDD) among sub-groups based on DSM-IV nicotine dependence (ND) status^a^.

Outcome	Non-nicotine dependent			Nicotine dependent		
MDD	(N = 618)			(N = 678)		
Risk Factor	OR	95% CI	p-value	OR	95% CI	p-value
rs2399496						
TT	1.00			1.00		
TA	1.47	0.80–2.70	0.214	1.19	0.76–1.88	0.450
AA	1.23	0.58–2.60	0.583	2.29	1.37–3.83	0.002

a Adjusted for sex, age and alcohol abuse.

To illustrate the relative contribution of the SNP and ND, we created a new variable combining ND status and number of rs2399496 minor alleles on individual level. Subjects with two rs2399496 minor alleles (AA) and ND (N = 165) had more than five-fold risk for lifetime MDD (OR 5.74, 95%CI 3.1–11, p = 9.0e-09) compared to subjects not fulfilling ND criteria and carrying no rs2399496 minor alleles (TT) (N = 177) (Table 5). We estimated the proportion of co-variation of ND and MDD accounted for by *DRD3* rs2399496. Based on logistic regression where MDD co-morbid with ND was the outcome variable rs2399496 explained 1.32% (Pseudo R^2^ = 0.0132) of co-variation. Finally, in order to verify that the ND diagnosis in our data reflects chronic exposure to cigarettes we conducted subgroup models, and detected significant association between rs2399496 and MDD among 673 heavy (ever) smokers (smoked ≥20 CPD) (OR_minor/minor_ = 2.44, 95% CI 1.45–4.10, p = 0.001). No association was detected among 575 non-heavy ever smokers.

**Table 5:** Logistic regressions on the associations of the *DRD3* variant rs2399496 and nicotine dependence (ND) with major depressive disorder (MDD): Models combining the ND (no/yes) and rs2399496 (TT = 0, TA = 1, or AA = 2 minor alleles) status.

	Adjusted model a		
	(N = 1296)		
	OR	95% CI	p-value
‘Effects’ of increasing number of risk factors			
no ND + TT	1.00		
no ND + TA	1.48	0.80–2.75	0.214
no ND + AA	1.24	0.58–2.64	0.581
ND + TT	2.50	1.30–4.83	0.006
ND + TA	3.01	1.72–5.28	0.00006
ND + AA	5.74	3.12–10.5	9.010e-09
‘Effect’ of nicotine dependence			
no ND + TT	1.00		
no ND + TA	0.81	0.38–1.72	0.581
no ND + AA	1.19	0.63–2.28	0.589
ND + TT	2.01	1.04–3.93	0.038
ND + TA	2.43	1.30–4.56	0.006
ND + AA	4.63	2.42–8.88	3.561e-06
‘Effect’ of the number of minor gene alleles			
ND + TT	1.00		
no ND + TT	0.40	0.21–0.77	0.006
no ND + TA	0.59	0.35–1.00	0.051
no ND + AA	0.49	0.25–0.96	0.038
ND + TA	1.20	0.77–1.89	0.421
ND + AA	2.29	1.38–3.82	0.001

a Adjusted for sex, age, and alcohol abuse.

## Discussion

Utilizing a Finnish sample of twins and their siblings ascertained for heavy smoking from the population based Finnish twin cohort, we aimed to scrutinize the association between lifetime DSM-IV diagnoses of MDD and ND, as well as the magnitude of genetic factors associated with this co-morbidity. Ever smokers with ND had over 2-fold risk for MDD compared to non-dependent ones, in concordance with earlier literature [2]. We detected significant association between rs2399496 1.5 kb downstream of *DRD3* and co-morbid MDD and ND. Rs2399496 is in high LD with rs3732790 (D’ = 1.0, r^2^ = 0.55), 274 bp downstream of *DRD3*, and with intronic rs2134655 (D’ = 0.98, r^2^ = 0.46). Rs3732790 showed similar trend, approaching suggestive association. As none of the variants have a clear functional role, we hypothesize that the detected association reflects LD with an unidentified functional variant. Although rs2399496 is imputed in the GWA data, its high minor allele frequency (0.47), results in improved imputation accuracy compared to rare variants. Individuals carrying two minor alleles had nearly 2-fold risk for lifetime MDD compared to individuals homozygous for the major allele. Although no association was detected between rs2399496 and ND, individuals with two minor alleles and ND diagnosis had over five-fold risk for lifetime MDD compared to individuals not fulfilling DSM-IV ND criteria and carrying no minor alleles.

Our results do not substantiate pleiotropic associations of *DRD3*, but rather support the gene-by-ND-interaction hypothesis, with ND enhancing the influence of rs2399496 on MDD risk. As portrayed in our second hypothesis, chronic heavy exposure to nicotine, sustained by ND, can be deemed as an environmental factor in the etiology of MDD. Supporting this, subgroup models among individuals with ND diagnosis yielded similar results than those among heavy ever smokers. Thus, in our data ND diagnosis seemed to reflect chronic nicotine exposure, although the DSM-IV ND criteria focus on other aspects of ND than smoking quantity. Gene-by-environment-interaction has been previously reported for *DRD2* and *DRD4* [17], [18]. In discordance with previous reports, we detected no association between the tested traits and *DRD2* rs1800497 (TaqIA) or any of the other *DRD2* SNPs. Similarly, no association was detected between *DRD4* variants and the included traits; however, the most commonly implicated *DRD4* 48-base-pair-repeat polymorphism was not assessed in the current study. We have the *DRD4* 48-bp minisatellite genotype data available in a subset of the study sample (N = 651); no association was detected with any of the phenotypes (data not shown).

Best evidence for replication was seen in the Dutch NTR-NESDA sample with the MDD-ND co-morbid phenotype, with a SNP in high LD with the SNP showing significant association in the study sample. The detected association in the Dutch sample may tag either the same or different underlying functional variant than in the Finnish sample. It is highly plausible that the underlying LD structure varies between the Finnish and Dutch populations, especially when considering the unique genetic architecture of Finns [42]. Population-specific functional variants are known to exist, and one has already been documented in the Finnish population for a behavioral trait [43]. Future studies are needed to expose whether rs2399496 in the Finnish study sample and rs3732790 in the Dutch NTR-NESDA sample tag the same functional variant. In concordance with the results obtained from the study sample, no statistically significant direct association was seen with ND in the NTR-NESDA sample. The association did not replicate in the Finnish adolescent FT12 sample or adult population sample or in the Australian twin family sample (NAG-AUS). In the adolescent sample DSM-IV ND diagnosis was not available, so FTND was used instead. DSM-IV predominantly measures loss of control in smoking behavior [44] while FTND measures physical dependence [45]. Concerning MDD, the FT12 sample was interviewed at an average age of 21.9 (SD 0.8, range 21–26). Prevalence of lifetime MDD was 12%, comparable to studies reporting 15–20% of youth experiencing a MDD episode by age 20 [1]. However, the core phenotype in genetic analyses was co-morbidity of MDD and ND with prevalence of only 2.8% (N = 38) in the FT12 sample. Thus, those analyses suffered from lack of power. In the population-based Health2000 sample MDD and ND DSM-IV diagnoses were not available; rather, we investigated associations with depression phenotypes defined by the CIDI for major depressive episode [23], and the BDI, a 21-question multiple-choice self-report inventory measuring severity of depression. In an attempt to create a phenotype resembling co-morbid MDD and ND, we examined depression phenotypes among heavy (≥20 CPD) ever smokers. The lack of association may partly reflect inappropriate phenotype definitions, as we had no means to identify the ‘extreme’ individuals with DSM-IV diagnoses for both MDD and ND. Lack of association in the Australian sample, despite availability of identical phenotypes, may reflect population specificity of the detected risk variant.

Our results expand the existing knowledge on the etiology of MDD and ND. It is plausible that the scarcity of association findings for *DRD3* and for the other *dopamine receptor* genes is partly due to complexity of the underlying mechanisms and inability to capture the signal when investigating one phenotype at a time. To date, single studies have identified specific *DRD3* variants associating with FTND defined ND [46], heaviness of smoking index, and time to first cigarette in the morning [11], as well as treatment response and remission in depression patients [47].

Converging pharmacological, post-mortem, and genetic data have suggested involvement of *DRD3* in drug dependence. Rather than being involved in direct reinforcing effects of drugs of abuse, *DRD3* appears to be implicated in motivation to self-administer drugs under schedules where response requirements are high [48]. A 30% reduction of *DRD3* expression in peripheral blood lymphocytes has been reported in current smokers compared to controls with no lifetime regular smoking, *DRD3* expression correlating negatively with CPD [49]. Given the known involvement of *DRD3* in reward mediation, such selective inhibiting effect of smoking on *DRD3* expression indicates vicious-cycle explanation of motivation for continued smoking [49]. Dysfunction of dopamine D3 receptors has also been linked to the pathogenesis of major depression [3]. Preclinical data show enhanced D3 receptor binding in the striatum upon antidepressant medication and electroconvulsive therapy [47].

We diligently addressed multiple testing. As the included markers and traits are correlated, standard procedures of correcting for multiple testing would be overly conservative. Thus, we used modified Bonferroni correction and utilized estimated numbers of independent markers and traits to set p-value thresholds for significant and suggestive associations. Estimation of independent markers, based on LD matrixes, is straightforward; however, estimating the number of independent traits is more challenging. We used a statistical estimate based on the correlation/covariance matrix, resulting in a sample-based estimate that may vary in novel independent population samples. Using estimated numbers of independent markers and traits in adjusting p-value thresholds is still quite conservative but nevertheless successful in reducing type I errors.

Although our sample size is moderate it is significantly larger than in most previous candidate gene studies addressing *dopamine receptor* genes and ND or depression. Our data on twins and siblings were ascertained specifically for smoking, the initial sample being drawn from the population-based Finnish twin cohort with extensive phenotypic profiles. Due to enrichment for ND (52% in the study sample *vs.* 40% in the Finnish population) our sample is also enriched for commonly co-occurring depression (17–18% in the study sample *vs.* 8–13% in the general population) yielding more power than presumed based on sample size. With adequate numbers of affected individuals available, we were able to focus on the most extreme phenotypes, *i.e*. DSM-IV diagnoses of ND and MDD, instead of investigating non-diagnostic phenotypes such as CPD and number of depressive symptoms. Although considered more powerful per se, neither of the quantitative DSM-IV symptom counts proved more informative than the corresponding dichotomous DSM-IV diagnoses. This is not surprising, as trait distributions in our enriched sample do not correspond with the population-level trait variance. Individuals with the most extreme phenotypes are likely to possess the most predisposing genetic variants [50] thus being most informative in genetic association analyses. Furthermore, the Finnish population represents a well-established isolate with minuscule population admixture. In isolates, genetic drift may lead to overabundance of morbid alleles for particular disorders and high proportion of patients is likely to share these alleles IBD. Although the association is strongest for rare disease alleles, isolates are also advantageous for genetic studies of common disorders [51]. Further, we should note that it is likely that our samples under study are relatively homogeneous being from the Finnish population, with little risk of bias from population stratification.

In this study where MDD-ND phenotype was the outcome variable rs2399496 explained 1.32% of the variance. This level of explanation is comparable to the finding of three genome-wide association (GWA) studies which reported variation in 15q24-25, containing three nAChR genes (*CHRNA5*, *CHRNA3*, *CHRNB4*), contributing to lung cancer risk and associating strongly with amount of smoking and ND [52]–[54] and where less than 1% of the variance in number of daily cigarettes smoked was explained by alleles of these genes.

To conclude, we detected a significant association between *DRD3* rs2399496 and MDD-ND co-morbid phenotype. We further demonstrated that ND strengthens the influence of the genetic variant on MDD, suggestive of gene-by-environment-interaction. We could not provide significant replication for these findings.

## Supporting Information

File S1 **Figure S1 and Tables S1–S4.** Figure S1. A) *DRD3* gene structure, B) genotyped SNPs, C) D' in the HapMap CEPH data (NCBI Build 36), D) r^2^ in the HapMap CEPH data, E) D' in the study sample (non-related individuals; one per family), F) r^2^ in the study sample. Table S1. Correlations between the included phenotypes. Correlations were computed by polychoric (tetrachoric and point biserial) and spearman correlation. Number of individuals varies from 1326 to 1428 depending on presence of missing values. Table S2. Marker quality controls. Table S3. Association analysis results (p-values) for all *dopamine receptor* genes. The study-specific P-value threshold for significant and suggestive association is 0.00042 and 0.0014, respectively. Table S4. a. The association of rs2399496, rs3732790 and rs2134655 with nicotine dependence (ND) and Major Depressive Disorder (MDD) in the Australian NAG-OZALC sample. Age, sex, and principal components (for population stratification) were used as covariates. All results are based on recessive models. b. The associations of rs2399496, rs3732790 and rs2134655 with nicotine dependence (ND) and Major Depressive Disorder (MDD) in the NTR-NESDA sample. Age, sex, and principal components (for population stratification) were used as covariates. All results are based on recessive models. c. The associations of rs2399496, rs3732790 and rs2134655 with nicotine dependence (ND) and Major Depressive Disorder (MDD) in the FT12 sample. Age and sex were used as covariates. All results are based on recessive models. d. The associations of rs2399496, rs3732790 and rs2134655 with nicotine dependence (ND^1^) and Major Depressive Disorder (MDD) in the T2000 sample. Age and sex were used as covariates. All results are based on recessive models.
(DOCX)
