# Supplementary material for: Role of Nicotine Dependence in the Association between the Dopamine Receptor Gene DRD3 and Major Depressive Disorder
Source: PLoS One. 2014 Jun 13;9(6):e98199. doi: 10.1371/journal.pone.0098199 (PMC4057087; doi:10.1371/journal.pone.0098199)
Supplement: File S1 — Figure S1 and Tables S1–S4. Figure S1. A) DRD3 gene structure, B) genotyped SNPs, C) D' in the HapMap CEPH data (NCBI Build 36), D) r2 in the HapMap CEPH data, E) D' in the study sample (non-related individuals; one per family), F) r2 in the study sample. Table S1. Correlations between the included phenotypes. Correlations were computed by polychoric (tetrachoric and point biserial) and spearman correlation. Number of individuals varies from 1326 to 1428 depending on presence of missing values. Table S2. Marker quality controls. Table S3. Association analysis results (p-values) for all dopamine receptor genes. The study-specific P-value threshold for significant and suggestive association is 0.00042 and 0.0014, respectively. Table S4. a. The association of rs2399496, rs3732790 and rs2134655 with nicotine dependence (ND) and Major Depressive Disorder (MDD) in the Australian NAG-OZALC sample. Age, sex, and principal components (for population stratification) were used as covariates. All results are based on recessive models. b. The associations of rs2399496, rs3732790 and rs2134655 with nicotine dependence (ND) and Major Depressive Disorder (MDD) in the NTR-NESDA sample. Age, sex, and principal components (for population stratification) were used as covariates. All results are based on recessive models. c. The associations of rs2399496, rs3732790 and rs2134655 with nicotine dependence (ND) and Major Depressive Disorder (MDD) in the FT12 sample. Age and sex were used as covariates. All results are based on recessive models. d. The associations of rs2399496, rs3732790 and rs2134655 with nicotine dependence (ND1) and Major Depressive Disorder (MDD) in the T2000 sample. Age and sex were used as covariates. All results are based on recessive models. (DOCX) [file pone.0098199.s001.docx]

**Figure S1.** A) *DRD3* gene structure, B) genotyped SNPs, C) D' in the HapMap CEPH data (NCBI Build 36), D) r^2^ in the HapMap CEPH data, E) D' in the study sample (non-related individuals; one per family), F) r^2^ in the study sample.

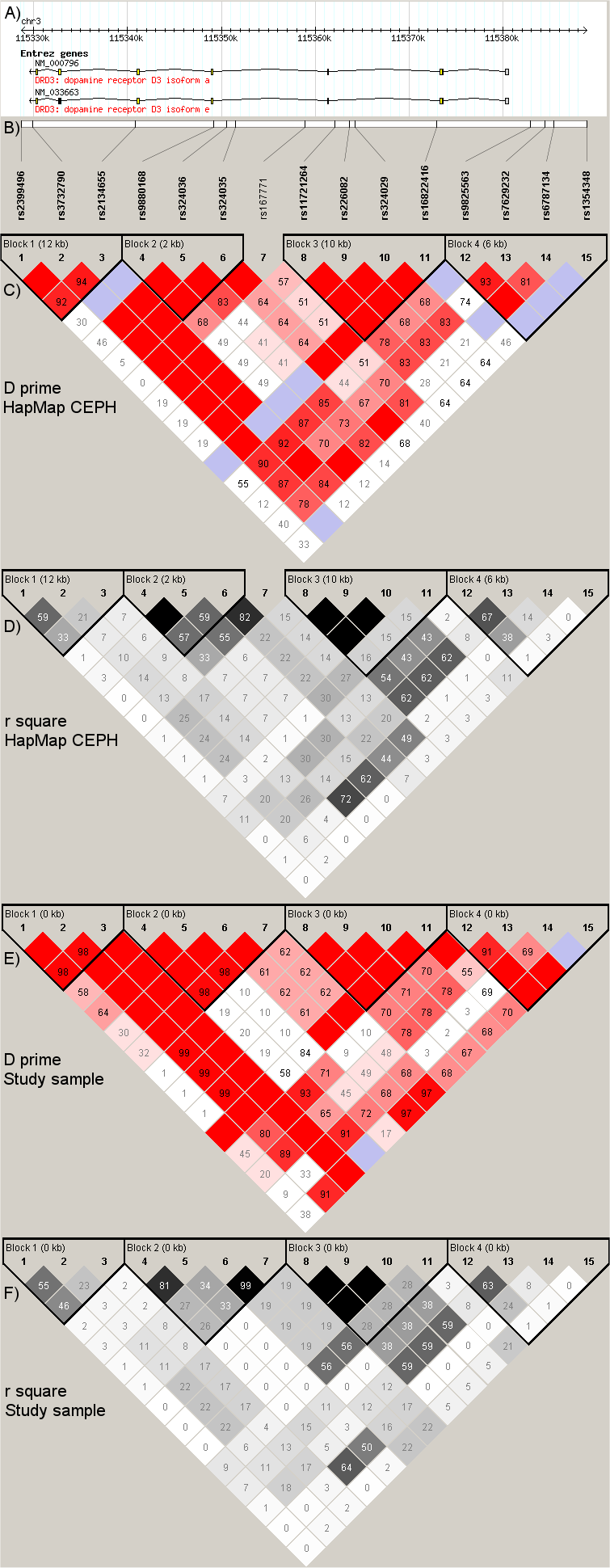
**Table S1.** Correlations between the included phenotypes. Correlations were computed by polychoric (tetrachoric and point biserial) and spearman correlation. Number of individuals varies from 1326 to 1428 depending on presence of missing values.

|  | DSM-IV MDD | DSM-IV MDD symptoms | DSM-IV ND | DSM-IV ND symptoms | Co-morbidity of DSM-IV MDD and ND |
| --- | --- | --- | --- | --- | --- |
| DSM-IV MDD | 1 |  |  |  |  |
| DSM-IV MDD symptoms | 0.87 | 1 |  |  |  |
| DSM-IV ND | 0.32 | 0.24 | 1 |  |  |
| DSM-IV ND symptoms | 0.33 | 0.26 | 0.93 | 1 |  |
| Co-morbidity of DSM-IV MDD and ND | 0.99 * | 0.78 | 0.88 * | 0.59 | 1 |

* Tetrachoric correlation with continuation correction by R software

**Table S2.** Marker quality controls.

| **SNP** | **Chr** | **Position** | **Distance** | **Gene(s)** | **Location** | **Amino acid change** | **Success** | **HWE** | **MAF** | **Alleles** | **GWAS** |
| --- | --- | --- | --- | --- | --- | --- | --- | --- | --- | --- | --- |
| rs2399496 | 3q13.31 | 113846013 |  | *DRD3* | Downstream |  | 94.4 % | 0.2639 | 0.480 | T/A | Imputed |
| rs3732790 | 3q13.31 | 113847283 | 1270 | *DRD3* | Downstream |  | 95.4 % | 0.8517 | 0.360 | T/A | Imputed |
| rs2134655 | 3q13.31 | 113858201 | 10918 | *DRD3* | Intron |  | 99.9 % | 1 | 0.298 | G/A | Genotyped |
| rs9880168 | 3q13.31 | 113866551 | 8350 | *DRD3* | Intron |  | 99.2 % | 1 | 0.052 | A/G | Imputed |
| rs324036 | 3q13.31 | 113867862 | 1311 | *DRD3* | Intron |  | 99.4 % | 1 | 0.065 | C/G | Imputed |
| rs324035 | 3q13.31 | 113868854 | 992 | *DRD3* | Intron |  | 99.3 % | 0.3811 | 0.167 | C/A | Imputed |
| rs167771 | 3q13.31 | 113876275 | 7421 | *DRD3* | Intron |  | 99.7 % | 0.6911 | 0.170 | A/G | Genotyped |
| rs11721264 | 3q13.31 | 113879404 | 3129 | *DRD3* | Intron |  | 99.8 % | 0.8536 | 0.287 | G/A | Imputed |
| rs226082 | 3q13.31 | 113881013 | 1609 | *DRD3* | Intron |  | 97.1 % | 0.8590 | 0.288 | A/G | Genotyped |
| rs324029 | 3q13.31 | 113881623 | 610 | *DRD3* | Intron |  | 99.4 % | 0.8536 | 0.287 | G/A | Genotyped |
| rs16822416 | 3q13.31 | 113890310 | 8687 | *DRD3* | Intron |  | 99.8 % | 1 | 0.103 | G/A | Imputed |
| rs9825563 | 3q13.31 | 113900220 | 9910 | *DRD3* | Intron |  | 99.3 % | 0.0334 | 0.236 | A/G | Genotyped |
| rs7629232 | 3q13.31 | 113901818 | 1598 | *DRD3* | Intron |  | 98.6 % | 0.5403 | 0.286 | T/C | Imputed |
| rs6787134 | 3q13.31 | 113902749 | 931 | *DRD3* | Intron |  | 99.5 % | 0.0496 | 0.069 | A/C | Genotyped |
| rs1354348 | 3q13.31 | 113906226 | 3477 | *DRD3* | Intron |  | 99.2 % | 0.8798 | 0.045 | A/C | Imputed |
| rs10033951 | 4p16.1 | 9779580 |  | *DRD5* | Upstream |  | 98.3 % | 1 | 0.331 | C/T | Imputed |
| rs1850744 | 4p16.1 | 9790712 | 11132 | *DRD5* | Downstream |  | 99.4 % | 1 | 0.040 | C/T | Imputed |
| rs265969 | 5q35.2 | 174857623 |  |  | Intergenic |  | 99.3 % | 0.5987 | 0.298 | T/C | Imputed |
| rs17065021 | 5q35.2 | 174858052 | 429 | *DRD1* | Downstream |  | 99.9 % | 0.1057 | 0.193 | G/A | Genotyped |
| rs2453737 | 5q35.2 | 174858902 | 850 | *DRD1* | Downstream |  | 97.5 % | 0.7994 | 0.328 | T/C | Imputed |
| rs6881740 | 5q35.2 | 174859162 | 260 | *DRD1* | Downstream |  | 97.4 % | 0.9858 | 0.323 | C/G | Imputed |
| rs11742274 | 5q35.2 | 174859497 | 335 | *DRD1* | Downstream |  | 97.2 % | 0.3929 | 0.175 | C/G | Imputed |
| rs265971 | 5q35.2 | 174860411 | 914 | *DRD1* | Downstream |  | 99.9 % | 0.6963 | 0.297 | A/G | Genotyped |
| rs265972 | 5q35.2 | 174860516 | 105 | *DRD1* | Downstream |  | 97.2 % | 0.4939 | 0.475 | A/G | Imputed |
| rs265973 | 5q35.2 | 174860699 | 183 | *DRD1* | Downstream |  | 99.9 % | 0.3477 | 0.495 | G/A | Genotyped |
| rs265974 | 5q35.2 | 174861240 | 541 | *DRD1* | Downstream |  | 97.2 % | 0.0786 | 0.347 | A/G | Genotyped |
| rs265975 | 5q35.2 | 174862195 | 955 | *DRD1* | Downstream |  | 99.6 % | 0.1069 | 0.346 | C/T | Imputed |
| rs265977 | 5q35.2 | 174863257 | 1062 | *DRD1* | Downstream |  | 97.8 % | 0.3726 | 0.195 | C/T | Imputed |
| rs863126 | 5q35.2 | 174863348 | 91 | *DRD1* | Downstream |  | 94.7 % | 0.8160 | 0.308 | A/T | Imputed |
| rs11747728 | 5q35.2 | 174863502 | 154 | *DRD1* | Downstream |  | 99.9 % | 0.5805 | 0.117 | A/G | Genotyped |
| rs835541 | 5q35.2 | 174863576 | 74 | *DRD1* | Downstream |  | 86.0 % | 0.8644 | 0.461 | G/A | Imputed |
| rs835616 | 5q35.2 | 174863690 | 114 | *DRD1* | Downstream |  | 99.8 % | 0.2212 | 0.380 | G/A | Genotyped |
| rs835540 | 5q35.2 | 174863891 | 201 | *DRD1* | Downstream |  | 97.5 % | 0.7016 | 0.250 | A/G | Genotyped |
| rs11746641 | 5q35.2 | 174866091 | 2200 | *DRD1* | Downstream |  | 99.4 % | 0.0449 | 0.142 | A/C | Genotyped |
| rs12518222 | 5q35.2 | 174867169 | 1078 | *DRD1* | Downstream |  | 89.7 % | 1 | 0.190 | C/T | Imputed |
| rs4867798 | 5q35.2 | 174867899 | 730 | *DRD1* | 3' UTR |  | 90.6 % | 0.9169 | 0.335 | T/C | Imputed |
| rs686 | 5q35.2 | 174868700 | 801 | *DRD1* | 3' UTR |  | 100 % | 0.7487 | 0.383 | A/G | Genotyped |
| rs265981 | 5q35.2 | 174870902 | 2202 | *DRD1* | 5' UTR |  | 97.8 % | 0.6728 | 0.373 | G/A | Imputed |
| rs3758653 | 11p15.5 | 636399 |  | *DRD4 DEAF1* | Upstream  Downstream |  | 99.9 % | 0.1304 | 0.233 | A/G | Genotyped |
| rs17758 | 11p15.5 | 644325 | 7926 | *DRD4 DEAF1* | Downstream  3' UTR |  | 94.2 % | 1 | 0.024 | G/A | Imputed |
| rs2734849 | 11q23.2 | 113270160 |  | *ANKK1*  *DRD2* | MissenseDownstream | His490Arg | 99.6 % | 0.4141 | 0.488 | A/G | Genotyped |
| rs2734848 | 11q23.2 | 113270374 | 214 | *ANKK1 DRD2* | Synonymous Downstream |  | 100 % | 0.4019 | 0.138 | A/G | Genotyped |
| rs1800497 | 11q23.2 | 113270828 | 454 | *ANKK1 DRD2* | MissenseDownstream | Glu713Lys | 99.9 % | 1 | 0.209 | G/A | Genotyped |
| rs11214599 | 11q23.2 | 113271360 | 532 | *ANKK1 DRD2* | Downstream  Downstream |  | 99.7 % | 0.9360 | 0.203 | C/T | Imputed |
| rs11214601 | 11q23.2 | 113272762 | 1402 | *ANKK1 DRD2* | Downstream  Downstream |  | 99.9 % | 0.9360 | 0.203 | C/T | Imputed |
| rs2587550 | 11q23.2 | 113272925 | 163 | *ANKK1 DRD2* | Downstream  Downstream |  | 99.8 % | 0.5787 | 0.303 | A/G | Genotyped |
| rs12422191 | 11q23.2 | 113274010 | 1085 | *ANKK1 DRD2* | Downstream  Downstream |  | 100 % | 0.7773 | 0.075 | G/A | Genotyped |
| rs10891549 | 11q23.2 | 113278447 | 4437 | *ANKK1 DRD2* | Downstream  Downstream |  | 99.4 % | 0.2778 | 0.493 | T/C | Imputed |
| rs2234689 | 11q23.2 | 113278483 | 36 | *ANKK1 DRD2* | Downstream  Downstream |  | 99.9 % | 0.3807 | 0.137 | C/G | Imputed |
| rs1554929 | 11q23.2 | 113278764 | 281 | *ANKK1 DRD2* | Downstream  Downstream |  | 99.6 % | 0.2778 | 0.493 | C/T | Imputed |
| rs6279 | 11q23.2 | 113281073 | 2309 | *ANKK1 DRD2* | Downstream  3' UTR |  | 99.6 % | 0.5787 | 0.303 | C/G | Imputed |
| rs1124491 | 11q23.2 | 113282090 | 1017 | *DRD2* | Intron |  | 99.8 % | 0.9360 | 0.203 | G/A | Imputed |
| rs1079595 | 11q23.2 | 113282669 | 579 | *DRD2* | Intron |  | 99.1 % | 0.9360 | 0.203 | A/C | Imputed |
| rs6275 | 11q23.2 | 113283477 | 808 | *DRD2* | Synonymous |  | 99.7 % | 0.2300 | 0.286 | G/A | Imputed |
| rs2440390 | 11q23.2 | 113286878 | 3401 | *DRD2* | Intron |  | 99.9 % | 0.4900 | 0.097 | G/A | Genotyped |
| rs1079727 | 11q23.2 | 113289182 | 2304 | *DRD2* | Intron |  | 98.8 % | 0.9360 | 0.203 | A/G | Genotyped |
| rs2734833 | 11q23.2 | 113292920 | 3738 | *DRD2* | Intron |  | 99.1 % | 0.1758 | 0.468 | A/G | Imputed |
| rs1076562 | 11q23.2 | 113296008 | 3088 | *DRD2* | Intron |  | 100 % | 0.2115 | 0.264 | G/A | Imputed |
| rs7131440 | 11q23.2 | 113299910 | 3902 | *DRD2* | Intron |  | 98.9 % | 0.1884 | 0.472 | T/C | Imputed |
| rs17115583 | 11q23.2 | 113308902 | 8992 | *DRD2* | Intron |  | 99.6 % | 0.5682 | 0.167 | G/A | Imputed |
| rs11214606 | 11q23.2 | 113309869 | 967 | *DRD2* | Intron |  | 99.8 % | 1 | 0.033 | G/A | Genotyped |
| rs4648318 | 11q23.2 | 113313389 | 3520 | *DRD2* | Intron |  | 99.9 % | 0.2351 | 0.272 | A/G | Genotyped |
| rs17529477 | 11q23.2 | 113317067 | 3678 | *DRD2* | Intron |  | 99.6 % | 0.1565 | 0.162 | G/A | Genotyped |
| rs17601612 | 11q23.2 | 113317745 | 678 | *DRD2* | Intron |  | 91.5 % | 0.0247 | 0.192 | G/C | Imputed |
| rs4245147 | 11q23.2 | 113318007 | 262 | *DRD2* | Intron |  | 96.9 % | 0.1054 | 0.281 | T/C | Imputed |
| rs4245148 | 11q23.2 | 113320419 | 2412 | *DRD2* | Intron |  | 99.6 % | 0.3239 | 0.066 | C/T | Imputed |
| rs7131056 | 11q23.2 | 113329774 | 9355 | *DRD2* | Intron |  | 100 % | 0.7794 | 0.495 | A/C | Genotyped |
| rs1799978 | 11q23.2 | 113346351 | 16577 | *DRD2* | Intron/Upstream * |  | 100 % | 1 | 0.097 | T/C | Imputed |
| rs12364283 | 11q23.2 | 113346955 | 604 | *DRD2* | Upstream |  | 99.9 % | 0.9382 | 0.049 | A/G | Genotyped |
| rs10891556 | 11q23.2 | 113352761 | 5806 | *DRD2* | Upstream |  | 100 % | 1 | 0.191 | C/A | Genotyped |

| Notes: |  | | |  |  |
| --- | --- | --- | --- | --- | --- |
| Position: | Based on GRCh37/hg19 (Feb 2009); in base pairs | | |  |  |
| Distance: | From previous SNP; in base pairs | | |  |  |
| Location: | Based on UCSC Genome Browser on Human Feb. 2009 (GRCh37/hg19) Assembly | | |  |  |
| Success: | Success rate; calculated from whole study sample (n=1467) | | | |  |
| HWE: | Hardy-Weinberg p-value; calculated from independent individuals (one per family; n=734) | | | | |
| MAF: | Minor allele frequency; calculated from independent individuals (one per family; n=734) | | | | |
| Alleles: | Major allele presented first |  |  | |  |
| Data: | G, Genotyped; I, Imputed |  |  | |  |
| *: | Depending on the isoform |  |  | |  |

**Table S3**. Association analysis results (p-values) for all *dopamine receptor* genes. The study-specific P-value threshold for significant and suggestive association is 0.00042 and 0.0014, respectively.

| Marker | *Gene* | DSM-IV MDD | DSM-IV MDD symptoms | DSM-IV ND | DSM-IV ND symptoms | Co-morbidity of DSM-IV MDD and ND |
| --- | --- | --- | --- | --- | --- | --- |
| rs2399496 | *DRD3* | 0.00076 | 0.0017 | 0.0584 | 1.0000 | **0.000079** |
| rs3732790 | *DRD3* | 0.0087 | 0.0072 | 0.4822 | 0.2864 | 0.0105 |
| rs2134655 | *DRD3* | 0.1247 | 0.0374 | 0.6960 | 0.4826 | 0.0469 |
| rs9880168 | *DRD3* | 0.0716 | 0.3370 | 0.9960 | 0.5836 | 0.1943 |
| rs324036 | *DRD3* | 0.0982 | 0.3365 | 1.0000 | 1.0000 | 0.0889 |
| rs324035 | *DRD3* | 0.7594 | 0.7321 | 0.5326 | 0.6952 | 0.9102 |
| rs167771 | *DRD3* | 0.9985 | 0.6094 | 0.9846 | 0.9012 | 0.6483 |
| rs11721264 | *DRD3* | 0.8690 | 0.5291 | 0.3303 | 0.4644 | 0.8275 |
| rs226082 | *DRD3* | 0.6740 | 0.4648 | 0.4447 | 0.4604 | 0.9501 |
| rs324029 | *DRD3* | 0.7196 | 0.3810 | 0.3832 | 0.4373 | 0.8738 |
| rs16822416 | *DRD3* | 0.3038 | 0.8761 | 0.7402 | 0.6740 | 0.1150 |
| rs9825563 | *DRD3* | 0.1283 | 0.3108 | 0.9979 | 0.6293 | 0.2091 |
| rs7629232 | *DRD3* | 0.2075 | 0.2885 | 0.4226 | 0.9149 | 0.3328 |
| rs6787134 | *DRD3* | 0.9466 | 0.9375 | 0.9436 | 0.6778 | 0.9520 |
| rs1354348 | *DRD3* | 0.2918 | 0.6780 | 0.5920 | 0.0352 | 0.5559 |
| rs10033951 | *DRD5* | 0.5112 | 0.0818 | 0.9928 | 0.7801 | 0.6452 |
| rs1850744 | *DRD5* | 0.4362 | 0.5163 | 0.9680 | 0.4250 | 0.3982 |
| rs265969 | *DRD1* | 0.1710 | 0.8861 | 0.3383 | 0.6897 | 0.2934 |
| rs17065021 | *DRD1* | 0.9279 | 0.4950 | 0.5858 | 0.7670 | 0.7847 |
| rs2453737 | *DRD1* | 0.7880 | 0.3971 | 0.6305 | 0.8375 | 0.2165 |
| rs6881740 | *DRD1* | 0.6242 | 0.5348 | 0.7099 | 0.7575 | 0.2635 |
| rs11742274 | *DRD1* | 0.3947 | 0.9867 | 0.4802 | 0.7408 | 0.5342 |
| rs265971 | *DRD1* | 0.1632 | 0.9093 | 0.3561 | 0.7001 | 0.3178 |
| rs265972 | *DRD1* | 0.9395 | 1.0000 | 0.6669 | 0.8847 | 0.2648 |
| rs265973 | *DRD1* | 0.2364 | 0.6227 | 0.2816 | 0.3221 | 0.6356 |
| rs265974 | *DRD1* | 0.4813 | 0.8006 | 0.9660 | 0.5208 | 0.3617 |
| rs265975 | *DRD1* | 0.9611 | 0.8617 | 0.9720 | 0.6198 | 0.5059 |
| rs265977 | *DRD1* | 0.9675 | 0.7606 | 0.2497 | 0.7146 | 0.6761 |
| rs863126 | *DRD1* | 0.4556 | 0.8780 | 0.5279 | 0.9736 | 0.1989 |
| rs11747728 | *DRD1* | 0.0136 | 0.7018 | 0.4630 | 0.8405 | 0.2705 |
| rs835541 | *DRD1* | 0.4667 | 0.9409 | 0.1273 | 1.0000 | 0.2085 |
| rs835616 | *DRD1* | 0.6300 | 0.4508 | 0.5136 | 0.8770 | 0.1541 |
| rs835540 | *DRD1* | 0.0319 | 0.2006 | 0.8216 | 0.5087 | 0.7506 |
| rs11746641 | *DRD1* | 0.0023 | 0.8010 | 0.1051 | 0.7133 | 0.1104 |
| rs12518222 | *DRD1* | 0.3409 | 0.8191 | 0.2597 | 0.7022 | 0.2743 |
| rs4867798 | *DRD1* | 0.2649 | 0.8276 | 0.7096 | 0.6867 | 0.6084 |
| rs686 | *DRD1* | 0.3292 | 0.4103 | 0.6884 | 0.5189 | 0.2326 |
| rs265981 | *DRD1* | 0.2751 | 0.3574 | 0.7563 | 0.7085 | 0.2824 |
| rs3758653 | *DRD4* | 0.4384 | 0.9604 | 0.1216 | 0.0951 | 0.3849 |
| rs17758 | *DRD4* | 0.1693 | 0.8642 | 0.0757 | 0.6453 | 0.1314 |
| rs2734849 | *DRD2* | 0.1637 | 0.8745 | 0.5129 | 0.8362 | 0.3257 |
| rs2734848 | *DRD2* | 0.9759 | 0.7531 | 0.9750 | 0.9387 | 0.6344 |
| rs1800497 | *DRD2* | 0.1288 | 0.6631 | 0.4727 | 0.7468 | 0.0988 |
| rs11214599 | *DRD2* | 0.2648 | 0.8582 | 0.9447 | 0.6782 | 0.2025 |
| rs11214601 | *DRD2* | 0.2627 | 0.8775 | 0.9413 | 0.6905 | 0.2015 |
| rs2587550 | *DRD2* | 0.5516 | 0.9523 | 0.9862 | 0.4955 | 0.9889 |
| rs12422191 | *DRD2* | 0.8329 | 0.7607 | 0.6071 | 0.5492 | 0.4175 |
| rs10891549 | *DRD2* | 0.3636 | 0.8731 | 0.6389 | 0.7529 | 0.5299 |
| rs2234689 | *DRD2* | 0.9944 | 0.5781 | 0.9808 | 0.7594 | 0.3931 |
| rs1554929 | *DRD2* | 0.2723 | 0.8692 | 0.6118 | 0.8058 | 0.5179 |
| rs6279 | *DRD2* | 0.5694 | 0.9108 | 0.9821 | 0.4648 | 0.9870 |
| rs1124491 | *DRD2* | 0.2216 | 0.8015 | 0.7799 | 0.7128 | 0.1762 |
| rs1079595 | *DRD2* | 0.2874 | 0.8308 | 0.9498 | 0.6203 | 0.2022 |
| rs6275 | *DRD2* | 0.5760 | 0.9597 | 0.7587 | 0.3477 | 0.9240 |
| rs2440390 | *DRD2* | 0.8213 | 0.5651 | 0.7050 | 0.6899 | 0.3807 |
| rs1079727 | *DRD2* | 0.1647 | 0.7036 | 0.9432 | 0.8004 | 0.1416 |
| rs2734833 | *DRD2* | 0.2392 | 0.8305 | 0.9790 | 0.5494 | 0.4036 |
| rs1076562 | *DRD2* | 0.5177 | 0.8441 | 0.6136 | 0.3509 | 0.9370 |
| rs7131440 | *DRD2* | 0.1801 | 0.8075 | 0.7819 | 0.6307 | 0.3994 |
| rs17115583 | *DRD2* | 0.4311 | 0.9176 | 0.4795 | 0.5033 | 0.5541 |
| rs11214606 | *DRD2* | 0.5525 | 0.8243 | 0.4043 | 0.1273 | 0.2267 |
| rs4648318 | *DRD2* | 0.4423 | 0.9631 | 0.9647 | 0.4819 | 0.9906 |
| rs17529477 | *DRD2* | 0.3846 | 0.2414 | 0.5333 | 0.4639 | 0.7962 |
| rs17601612 | *DRD2* | 0.3761 | 0.1736 | 0.8401 | 0.9193 | 0.1966 |
| rs4245147 | *DRD2* | 0.4063 | 0.5617 | 0.1988 | 0.2300 | 0.6641 |
| rs4245148 | *DRD2* | 0.6689 | 0.1017 | 0.1430 | 0.0320 | 0.7272 |
| rs7131056 | *DRD2* | 0.4920 | 0.4053 | 0.8631 | 0.9504 | 0.2277 |
| rs1799978 | *DRD2* | 0.6115 | 1.0000 | 0.7194 | 0.8504 | 0.6172 |
| rs12364283 | *DRD2* | 0.4442 | 0.6660 | 0.5698 | 0.6855 | 1.0000 |
| rs10891556 | *DRD2* | 0.8647 | 0.8804 | 0.1953 | 0.6683 | 0.7627 |

DSM-IV, Diagnostic and Statistical Manual of Mental Disorders, 4^th^ edition (APA 1994); MDD, major depressive disorder; ND, nicotine dependence

**Table S4a.** The association of rs2399496, rs3732790 and rs2134655 with nicotine dependence (ND) and Major Depressive Disorder (MDD) in the Australian NAG-OZALC sample. Age, sex, and principal components (for population stratification) were used as covariates. All results are based on recessive models.

| Marker | MAF (minor/major allele) | Association with DSM-IV ND (N affected = 1745) | | | Association with DSM-IV MDD  (N affected = 1268) | | | Association with DSM-IV MDD among ever smokers  (N affected = 1198) | | | Association with co-morbid DSM-IV MDD and ND  (N affected = 629) | | |
| --- | --- | --- | --- | --- | --- | --- | --- | --- | --- | --- | --- | --- | --- |
|  |  | OR | 95% CI | P | OR | 95% CI | P | OR | 95% CI | P | OR | 95% CI | P |
| rs9817063^1^ | 0.49 (C/T) | 0.89 | 0.74-1.06 | 0.194 | 1.06 | 0.90-1.24 | 0.480 | 1.04 | 0.88-1.23 | 0.630 | 0.96 | 0.77-1.19 | 0.721 |
| rs3732790 | 0.34 (A/T) | 0.92 | 0.70-1.21 | 0.542 | 1.12 | 0.90-1.40 | 0.320 | 1.10 | 0.87-1.40 | 0.432 | 1.09 | 0.80-1.48 | 0.575 |
| rs2134655 | 0.19 (T/C) | 1.03 | 0.44-2.39 | 0.944 | 1.05 | 0.50-2.23 | 0.894 | 0.77 | 0.37-1.61 | 0.489 | 0.87 | 0.29-2.58 | 0.804 |

**^1^** proxy of rs2399496

**Table S4b.** The associations of rs2399496, rs3732790 and rs2134655 with nicotine dependence (ND) and Major Depressive Disorder (MDD) in the NTR-NESDA sample. Age, sex, and principal components (for population stratification) were used as covariates. All results are based on recessive models.

| Marker | MAF  (minor/major allele) | Association with ND^1^  (N affected = 561) | | | Association with  DSM-IV MDD  (N affected = 1617) | | | Association with DSM-IV MDD among ever smokers^2^  (N affected =1200) | | | Association with co-morbid DSM-IV MDD and ND^3^  (N affected = 310) | | |
| --- | --- | --- | --- | --- | --- | --- | --- | --- | --- | --- | --- | --- | --- |
|  |  | OR | 95% CI | P | OR | 95% CI | P | OR | 95% CI | P | OR | 95% CI | P |
| rs2399496 | 0.49 (A/T) | 0.90 | 0.69-1.16 | 0.394 | 0.93 | 0.79-1.10 | 0.414 | 0.86 | 0.70-1.06 | 0.154 | 0.83 | 0.58-1.18 | 0.294 |
| rs3732790 | 0.41 (A/T) | 1.17 | 0.88-1.55 | 0.284 | 1.13 | 0.93-1.36 | 0.214 | 1.19 | 0.94-1.52 | 0.153 | 1.56 | 1.05-2.33 | 0.028 |
| rs2134655 | 0.27 (T/C) | 1.09 | 0.72-1.66 | 0.669 | 0.89 | 0.68-1.17 | 0.403 | 0.79 | 0.57-1.11 | 0.179 | 0.92 | 0.52-1.63 | 0.766 |

^1^ Fagerstrom Test for Nicotine Dependence (FTDN) score ≥4

^2^ Ever smokers (current+former)

^3^ MDD cases with FTND score ≥4

**Table S4c.** The associations of rs2399496, rs3732790 and rs2134655 with nicotine dependence (ND) and Major Depressive Disorder (MDD) in the FT12 sample. Age and sex were used as covariates. All results are based on recessive models.

| Marker | MAF (minor/major allele) | Association with ND^1^  (N affected = 167) | | | Association with DSM-IV MDD  (N affected = 105) | | | Association with DSM-IV MDD among ever smokers^2^  (N affected = 65) | | | Association with co-morbid DSM-IV MDD and ND^3^  (N affected = 28) | | |
| --- | --- | --- | --- | --- | --- | --- | --- | --- | --- | --- | --- | --- | --- |
|  |  | OR | 95% CI | P | OR | 95% CI | P | OR | 95% CI | P | OR | 95% CI | P |
| rs2399496 | 0.47 (A/T) | 0.71 | 0.46-1.11 | 0.134 | 0.75 | 0.43-1.32 | 0.318 | 0.58 | 0.28-1.21 | 0.148 | 0.45 | 0.13-1.55 | 0.209 |
| rs3732790 | 0.36 (A/T) | 0.82 | 0.45-1.47 | 0.501 | 0.78 | 0.39-1.54 | 0.480 | 0.48 | 0.17-1.35 | 0.162 | 0.28 | 0.04-2.11 | 0.217 |
| rs2134655 | 0.28 (T/C) | 0.80 | 0.41-1.57 | 0.526 | 0.57 | 0.22-1.46 | 0.240 | 0.56 | 0.17-1.82 | 0.335 | -^4^ | - | - |

^1^ FTND score ≥4

^2^ Ever smokers (current+former)

^3^ MDD cases with FTND score ≥4

^4^ Not estimated because of 0 cases in one cell

**Table S4d.** The associations of rs2399496, rs3732790 and rs2134655 with nicotine dependence (ND^1^) and Major Depressive Disorder (MDD) in the T2000 sample. Age and sex were used as covariates. All results are based on recessive models.

| Marker | MAF (minor/major allele) | Association with DSM-IV ND^1^ (N affected = 238) | | | Association with DSM-IV MDD  (N affected = 108) | | | Association with DSM-IV MDD among ever smokers^2^  (N affected = 61) | | | Association with co-morbid DSM-IV MDD and ND^3^  (N affected = 10) | | |
| --- | --- | --- | --- | --- | --- | --- | --- | --- | --- | --- | --- | --- | --- |
|  |  | OR | 95% CI | P | OR | 95% CI | P | OR | 95% CI | P | OR | 95% CI | P |
| rs2399496 | 0.44 (A/T) | 1.16 | 0.83-1.62 | 0.378 | 0.87 | 0.53-1.42 | 0.584 | 0.80 | 0.41-1.56 | 0.506 | 1.62 | 0.42-6.31 | 0.486 |
| rs3732790 | 0.40 (A/T) | 1.25 | 0.87-1.86 | 0.208 | 0.99 | 0.55-1.77 | 0.976 | 0.79 | 0.35-1.78 | 0.573 | -^4^ | - | - |
| rs2134655 | 0.28 (T/C) | 1.03 | 0.61-1.73 | 0.912 | 1.25 | 0.65-2.38 | 0.505 | 1.18 | 0.49-2.84 | 0.719 | 1.13 | 0.14-8.99 | 0.910 |

^1^ Heavy smoking used as a proxy of ND

^2^ Ever smokers (current+former)

^3^ MDD cases who are heavy smokers (proxy for ND)

^4^ Not estimated because of 0 cases in one cell
